# Latest Developments in the Management of Nut Allergies

**DOI:** 10.1007/s40521-021-00290-2

**Published:** 2021-06-15

**Authors:** H. A. Brough, R. Gourgey, S. Radulovic, J. C. Caubet, G. Lack, A. Anagnostou

**Affiliations:** 1 Paediatric Allergy Group, Department Women and Children’s Health, School of Life Course Sciences, King’s College London, London, UK; 2 Paediatric Allergy Group, School of Immunology and Microbial Sciences, King’s College London, London, UK; 3 Children’s Allergy Service, Evelina Children’s Hospital, Guy’s and St. Thomas’s NHS Foundation Trust, 2nd Floor, Stairwell B, South Wing, Westminster Bridge Rd, London, SE1 7EH UK; 4 Section of Allergy, Immunology and Retrovirology, Baylor College of Medicine, Houston, TX USA; 5 Department of Paediatric Allergy, The Royal London Children’s Hospital, Barts Health NHS Trust, E1 1FR, London, UK; 6 Pediatric Allergy Unit, University Hospitals of Geneva and University of Geneva, Rue Willy Donzé, Geneva, Switzerland; 7 Department of Pediatrics, Section of Allergy, Immunology and Retrovirology, Texas Children’s Hospital, Houston, TX USA

**Keywords:** Nut allergy, Dietary management, Epinephrine, Food immunotherapy

## Abstract

**Purpose of review:**

In this review, we sought to describe the most recent advances in the dietary and medical management of peanut and tree nut allergy, including selective introduction and immunotherapy.

**Recent findings:**

Dietary updates include changes to labeling laws, improved information sources, and new apps for buying foods in shops and overseas to better protect individuals with nut allergies. There are still issues in the management of nut allergies in schools, such as parents having to resort to packed lunches instead of school meals and patients experiencing bullying. Air travel also poses concern, but additional resources are now available to travelers, and recent evidence suggest limited airborne exposure to nuts. The medical management of anaphylaxis is use of epinephrine; however, this remains underutilized. Needle length and administration devices have been recently debated considering the risk of bone penetration vs subcutaneous administration, and autoinjectors seem to deliver higher peak concentrations than syringes. Selective nut introduction has gained momentum in the last 5 years, demonstrating improved quality of life but with the need for motivated parents for continued consumption and available resources for challenges. Immunotherapy to nuts is also a rapidly developing field, with the balance of efficacy and safety being important considerations in the differing modes of administration.

**Summary:**

The management of nut allergies is a rapidly developing field, and dietary and medical management have progressed significantly in the last 5 years. Future research directions include improving safety and efficacy of food immunotherapy and examining patients’ goals for therapy and treatment outcomes.

## Introduction

Nuts are increasingly consumed worldwide and include mainly tree nuts (TN), which refer to any nuts coming from a tree, as opposed to peanut belonging to the legume family [1]. Peanut and TN allergies have been increasingly reported since the 1980s and constitute currently a major public health concern. The prevalence of peanut/TN allergy ranges between 0.05 and 4.9% [2]. The severity of reactions and the impact on quality of life are important characteristics of nut allergy [3, 4]. Thus, an accurate diagnosis of patients with a suspicion of nut allergy is essential. The diagnosis is often made on the basis of patient’s history in conjunction with the results of skin prick tests (SPTs), specific IgE, and component resolved diagnostics (CRD) [5]. Clinical history, however, is not always clear, and it may be hard to establish the type of nut that triggered the reaction. The oral food challenge (OFC) has remained instrumental in establishing an accurate and specific diagnosis of peanut and tree nut allergies [6, 7] ;however, there are developments in the field of CRD and basophil activation testing to reduce the need for OFCs in certain patients as well as for the prediction of severity of clinical reactions [8].

The cornerstone of management of patients with nut allergy has been avoidance of the incriminated nut as well as other potentially related nuts. More recently, introduction of other nuts following a negative allergic workup in a patients with a given nut allergy has been discussed [9]. An optimal nutritional support is of major importance in the management of nut allergy and especially selective nut introduction. Only a small proportion of nut allergic patients will outgrow their allergy [10]. As an alternative to avoidance, food immunotherapy has been studied mainly for peanut, but also for tree nuts [11]. In this review, we will discuss the up-to-date management and treatment of patients with primary peanut/TN allergy, focusing on recent evidence and literature. Of note, in this review, we will not discuss pollen-related nut allergies, such as those linked to birch pollen allergy and PR-10 proteins.

## Dietary management

The dietary management of nut allergies includes allergen avoidance, use of relevant information resources, knowledge of labeling laws, and precautionary labeling and navigating daily life activities such as traveling and eating out.

Careful avoidance of nuts to which the person is allergic to is currently the mainstay of dietetic management. This involves education to ensure that the patient/caregiver understands what constitutes the allergic nut(s) and what food/products may contain these. Communicating that peanuts can also be called monkey nut, groundnut and earth nut and tree nuts include almond, hazelnut, cashew, pistachio, pecan, macadamia/Queensland nut, Brazil and walnut is essential. It is important to be aware that terminology varies in different countries and in the US foods including coconut, pine nut, and nutmeg which are classified as a tree nut, whereas they are not considered a tree nut in the UK and the EU. Education should be tailored to individual language and literacy skills, and pictorial forms of nuts should be shown and shared [12]. Discussion should also include recognizing nuts can be hidden in foods, and nuts are in non-food products including soaps, body lotions, and creams. Allergy-trained dietitians are well placed to provide this advice.

Information sources used by healthcare professionals in the UK include a Food Allergy Specialist Group (FASG) leaflet available to dietitians through the British Dietetic Association (BDA). Allergy UK and Anaphylaxis Campaign have open access resources for patients. Webinars are also available on nut avoidance (Allergy Academy, allergyacademy.org). In the USA, FARE (Food Allergy Research and Education, www.foodallergy.org) provides patient support and multiple relevant resources. Social media is often used by patients, though mostly for safety updates, according to a recent Spanish cross-sectional survey of 193 patients (guardians (n=162) and adolescents (n=31), in a tertiary hospital, where almost a half of respondents had nut allergy (47%). Sixty-seven percent of guardians used social media with 30% using it for food allergy–related inquiries and with 90% of adolescents using social media and only 7% using it for food allergy–associated intentions [13]. This may change over time, and particularly during the Covid-19 pandemic, health care professionals (HCPs) have been signposting patients to known reliable websites. Apps to help patients with buying foods free from nuts are also recommended as useful resources (such as Food Maestro©). The app Soose© can also translate into 15 languages. These apps use barcodes to scan the ingredients of the prepackaged food, and these can then be checked against a personal allergy profile. Currently there has not been any published clinical validation work around these apps.

Careful reading of food labels is recommended together with reminders that ingredient lists can change over time. The UK follows EU regulations with regard to allergen labeling with full ingredient lists and allergen labeling required for the 14 most common allergens on prepackaged foods and non-prepackaged foods including cafes and restaurants. Foods made and packaged on the premises were initially exempt from this law. However, following the death of Natasha Ednan-Laperouse, a new UK law [14] will come into effect in October 2021. All prepackaged food for direct sale to the public must now include a full list of ingredients with allergens highlighted. In the USA, only the 8 major allergens (milk, soybean, egg, wheat, fish, crustacean shellfish, peanut, tree nuts) are currently declared on all packaged foods.

A discussion around precautionary allergen labeling (PAL) with advice about avoiding all foods labeled with nut PAL is required for those with severe nut allergy. Providing advice around the higher risk foods such as chocolate, confectionery, and cereal bars provides more information for individuals when making a risk assessment. There is currently a voluntary system for PAL [15], resulting in consumer confusion as to the actual risk, and different terminologies are often used. A recent report by the European Federation of Allergy and Airways Diseases Patient Associations has called for a harmonized EU approach to PAL statements, establishment of reference doses for the 14 major allergens as well as risk assessment and recall [16]. Some clinicians have proposed that for peanut allergy sufferers, individual risk assessment could be undertaken with those passing a single 30 mg peanut protein dose challenge being allowed to ignore PAL. However, as recent research has shown that exercise and sleep deprivation can alter individuals’ threshold to peanut tolerance, this must be considered when preparing personalized risk assessment plans [17]. A recent pan European survey, of 1560 people living with food allergy, sought to assess their risk with PAL and how they prefer risks to be communicated. A total of 66% stated that a label combining a “statement + symbol” signifying a qualitative risk assessment would help with understanding the process used by the food manufacturer. If a qualitative risk assessment had been used in the decision about whether to include a PAL, 73% reported that it would improve their trust in the product [18].

Daily activities are often significantly impacted in food allergic patients. Eating out is considered a high-risk venture for those with peanut and tree nut allergy. In a Food Standards Agency 2018 survey of 2510 respondents, only just over half (53%) stated that they felt at all confident that food companies provide correct facts about allergens in food they provide [19]. A recent American report has provided guidance for improving legislation, education to restaurant staff, and HCP education [20]. Activities suggested to reduce accidental nut ingestion include (a) providing education about the type of high risk venues to be avoided (such as Asian restaurants and bakeries), (b) sharing both verbal and written facts with the venue (e.g., a “chef card” available on www.foodallergy.org in 10 languages), (c) eating at least busy times, and (d) always carrying emergency medication.

Empowering patients with nut allergy with as much knowledge as possible to help them make life less of a burden is essential. In addition, further public education is required as the investigators of the pan-European APPEAL-1 study concluded following their report of 1846 respondents, with peanut allergy (PA). Restrictions on important life choices were apparent including food choices (84–93%), going to parties and special occasions (89%), and choice of holiday destination (76%). Stress and frustration were voiced by 40% of respondents. Social isolation (66%) and bullying (43%) were also significant issues [21]. Addressing bullying during an allergist consultation was also highlighted in a recent survey from the American Academy of Allergy, Asthma and Immunology (AAAAI). It transpired that 7.3–8.3% of allergists were asking about bullying all the time, whereas 40–46% some of the time [22]. The issue of food allergy and bullying is becoming increasingly prevalent and is also highlighted in an Australian study, which included 93 children and adolescents, 70% of whom had peanut and 65% tree nut allergy. The investigators showed that 42% of respondents experienced bullying and in 23% this was related to their food allergy [23].

Air travel is another area of concern**.** Nut allergic patients and carers often ask about dietary advice when traveling by airplane. The key advice is to take one’s own food so as to reduce the risk of eating anything that may be cross-contaminated with nuts. Wiping the area around the seat and folding tray also helps with a recent small study finding detectable amounts of peanut protein on the surfaces of airplanes [24]. A recent review of environmental exposure to peanut from airborne sources reported that this is very unlikely to pose any significant risk [25]. Different airlines have different policies around preventing allergic reactions and Allergy Living has a guide to 16 airline policies on food allergies, a helpful patient resource [26].

Dietary management will include discussions around precautions to take in playgroups, nursery, reception, schools, and college. A clear allergy management plan naming the specific nut allergens to share with the organization and teachers is paramount. The plan will require sharing with those running extracurricular outings and events. In Venter et al.’s recent review of “management of peanut allergy” [27], an age-based list of recommendations is provided with an emphasis on the change in responsibility and knowledge being transferred from the carer/teacher to the child/adolescent. Consideration of wearing medical alert jewelry is also suggested. In a large qualitative review of 178 participants in the USA, one of the key themes to emerge was the lack of trust with school meals and needing to rely on food from home, which may place an additional load on those on lower incomes [28]. During the Covid-19 pandemic, there may be changes to school policies around mealtimes that may affect nut allergic children and create additional anxiety amongst a population that already has additional QoL burdens. However, activities such as hand-washing, cleaning areas, and not sharing food will help to mitigate risks from both spreading infection and managing food allergies to provide a safe place for all students with and without food allergy [29].

## Medical management

The medical management of peanut and tree nut allergy does not differ, in principle, from the management of other food allergies. It involves (a) acute management of an allergic reaction caused by exposure to the culprit nut and (b) long-term management, which currently relies mainly on avoidance strategies in order to minimize risk of any future reactions.

Peanut and tree nuts have remained an important cause of anaphylaxis and fatalities. A retrospective 15-year review of death records, analysis of emergency department visits (ED) and hospital admissions performed in New York City [30], identified peanuts to be the most common allergen implicated in hospitalization (27.1%) and ED visits (20.2%), followed closely by fish, tree nuts, and seeds. The same review identified peanuts to be the second most common cause of food-related anaphylaxis deaths in all ages. Those data correlate with other, earlier published data from other parts of the world. The analysis of the national anaphylaxis data showed that peanuts and tree nuts were the most common cause of deaths caused by food induced anaphylaxis, with peanuts and tree nuts being identified in 69/95 (73%) fatalities [31].

Epinephrine is the cornerstone and first-line treatment for nut-induced anaphylaxis, and patients at risk should have easy access to epinephrine autoinjectors (EAIs) in the community. Despite the clear guidelines of the European Academy of Allergy and Clinical Immunology (EAACI) [32] and the American Academy of Allergy, Asthma and Immunology (AAAAI) [33] on the importance of early recognition of anaphylaxis and prompt administration of epinephrine, EAIs have remained underutilized in both the community and hospital setting. According to a recent European Anaphylaxis Registry review of 10.184 reported cases of anaphylaxis, only 23.2% were treated with epinephrine. Patients presenting with anaphylaxis were more likely to be treated with epinephrine by a medical professional (27.1%) than a lay person (14.7%) [34]. A prospective 1-year study of 180 patients who presented at a single ED reported the use of epinephrine in only 25% of patients. Interestingly, epinephrine was more likely to be given by bystander and paramedics rather than a physician in the community or medical professionals at the hospital emergency department [35]. A literature review published by Wasserman et al. also highlighted a low rate of adrenaline use in the treatment of anaphylaxis. The same review also identified a low rate of prescription of epinephrine autoinjectors, even in patients who presented and were treated at the emergency department for anaphylaxis [36].

The standard recommended dose of epinephrine in anaphylaxis is 0.01 mg/kg for children weighing 30 kg or less, with a maximum dose of 0.3 mg for children and 0.5 mg for adults delivered intramuscularly. This recommendation is mainly extrapolated from doses used in other emergency situations, and there is still limited evidence to support it. Dosing options are currently limited to 0.15 and 0.3 mg worldwide, with the additional 0.1mg dose available in the USA for infants. The question of injection depth is particularly important when delivering epinephrine by autoinjectors, used in the community but also the hospital setting. Needle lengths in EAI devices have been the topic of ongoing discussions. Concerns regarding delivering sub-optimal dose or overdosing patients and potential risk of intraosseous or subcutaneous rather than intramuscular injection have remained. Brown et al. reported that using the 0.15mg epinephrine autoinjector (EAI) in children weighing less than 15 kg results in a dose in excess of 0.01mg/kg which increases to 150% excess in children weighing 10kg [37]. The American Academy of Pediatrics clinical report recommends switching to EAI 0.3mg when the child weighs 25kg or more [38]. The different needle lengths have also been studied in conjunction with pressure used to deliver medication by various commercially available EAIs. Dreborg et al. reported that using EAI 0.15mg in children weighing less than 15kg carried a potential risk of up to 60% of bone penetration, when calculated for use of the longest needle length accepted for a distribution. Interestingly this risk remained quite high at 29% in children weighing 15–30kg. However, this risk was negligible, when using low pressure EAI. Using the shortest needles for EAIs 0.3 mg in adolescents and adults carries a medium to high risk of subcutaneous injection, which reduces when using the longest needles approved for each device [39]. However, recent open-label, randomized, cross-over study investigating pharmacokinetics and pharmacodynamics of epinephrine 0.3 mg dose delivered by commercially available EAI or via intramuscular (IM) syringe into the anterolateral thigh in adults found that EAIs performed better in terms of peak adrenaline concentration, when compared to IM syringe ((0.52 vs 0.35 ng/mL). Epinephrine also reached maximum concentrations more rapidly after use of EAIs versus IM syringe, with a shorter median peak time (20 versus 50 min, respectively), but the overall exposure to epinephrine was similar [40].

Long-term management consists of correct identification of nut(s) the patient is allergic to and avoidance of the culprit nut(s). Establishing diagnosis of peanut/tree nut(s) allergies accurately has become particularly important in more recent years when the approach of avoidance strategies has changed from complete avoidance of all nuts to selective, patient-tailored nut avoidance. Recent studies such as Pronuts and NUTCRACKER showed that selective nut introduction is feasible and that it improves quality of life [9, 41]. In the Pronuts study, the median number of nut allergies was 2, and on average, children were able to introduce nine nuts or sesame seed into their diet [9]. However, performing sequential oral food challenges to determine allergy versus tolerance is labor and resource intensive, often requiring multiple visits for the family and is not without risk of severe allergic reactions. Strategies assuring either strict, blanket avoidance of all nuts in peanut and tree nuts allergic children or selective, patient-tailored, nut(s) avoidance requires shared decision-making and high motivation from the family as, after introduction, the selective nuts need to continue to be consumed regularly in the diet. A multidisciplinary approach including dietary advice to prevent cross-contact with the index nut, good education of patients, their families and other carers, and the facilities to perform oral food challenges is essential for this approach.

## Food immunotherapy

Over the last decade, food immunotherapy has emerged as a form of active and potentially disease-modifying treatment for common food allergies encountered in childhood. The process of immunotherapy involves the administration of small, gradually increasing doses of the food that patients are allergic to, with the aim to enable them to eat varying amounts of the allergenic food without reactions. There is a variety of terms used related to immunotherapy. “Desensitization” refers to a raise in the allergenic threshold of reactivity and implies regular dose ingestion. “Sustained unresponsiveness” refers to the ability of subjects who have completed an immunotherapy protocol, to take breaks off treatment (usually a few weeks/months) and then return to daily allergen consumption at their previous dose, without suffering any allergic reactions. “Long-term tolerance” is defined as the ability to eat the previously allergenic food ad lib (any amount and any period of food abstinence, however prolonged) without the need for daily dosing.

Food immunotherapy can be administered using different routes. The most studied is the oral (OIT; food is ingested), but sublingual (SLIT; application of food under the tongue), and epicutaneous (EPIT; application on the skin) have also been examined as alternatives. To date, most immunotherapy research trials have focused on one of the common childhood allergens such as cow’s milk, hen’s egg, peanut, and tree nuts.

A single-center walnut OIT trial has shown that walnut OIT can induce desensitization to walnut as well as cross-desensitization to pecan and hazelnut in patients who have tree nut co-allergies. In the trial, 49 (89%) of 55 patients in the oral immunotherapy group were desensitized to walnut compared with none of 18 patients in the control group. Following walnut desensitization, all patients who were co-allergic to pecan (*n*=46) were also desensitized to pecan. Additionally, 18 (60%) of 30 patients who were co-allergic to hazelnut or cashew, and 14 (93%) of 15 patients who were co-allergic to hazelnut alone, were either fully desensitized or responded to treatment [42]. Tree nuts are also often incorporated in multi-food OIT protocols. Different routes of food immunotherapy are associated with different efficacy and safety profiles (see Table 1).

**Table 1:** Comparison of dosing, duration, adverse events, and efficacy of desensitization and sustained unresponsiveness for oral immunotherapy (OIT), epicutaneous immunotherapy (EPIT), and sublingual immunotherapy (SLIT)

	OIT	EPIT	SLIT
Route of administration	Oral (ingestion)	Epicutaneous (applied on the skin)	Sublingual (under the tongue)
Foods	Peanut, tree nuts	Peanut	Peanut, tree nuts
Most commonly used daily dose	300 mg	250 μg	2–7 mg
Approximate time* to achieve initial desensitization	6–12 months	2–3 years	2–3 years
Desensitization efficacy	High	Small to moderate	Small to moderate
Adverse events	Local: frequent Anaphylaxis: infrequent	Local: frequent Anaphylaxis: rare	Local: frequent Anaphylaxis: rare
Sustained unresponsiveness	Variable	Not evaluated	Not evaluated
Quality of life post-intervention	Improved	Improved	Not evaluated

* Varies between different research studies

In OIT most subjects will likely experience mild or moderate reactions during treatment [43]. The frequency and number of reactions generally decrease during the maintenance phase, and it has been suggested that adverse events are significantly associated with allergic rhinitis and SPT wheal size [44]. The development of eosinophilic esophagitis (EoE) as a result of OIT is a current concern, but it is not clear whether OIT causes EoE or unmasks a pre-existing tendency. A published meta-analysis reports that approximately 3% of patients with IgE-mediated food allergies undergoing OIT developing this complication, with EoE often resolving following discontinuation of treatment [45]. However, individual studies have reported variable rates and the available data are limited. SLIT is administered in a liquid form, held under the tongue for a few minutes and then spat out or swallowed [46–50]. The typical starting dose for SLIT is lower than OIT (usually in micrograms rather than milligrams of food protein) as is the maintenance dose; therefore, SLIT is generally less effective when compared to OIT. The safety profile is also quite different, with uncommon systemic reactions, reported in up to 2.3% of doses [47–50]. Symptoms are typically mild and localized to the oropharyngeal region [51]. EoE has not been observed with food allergen SLIT but has been reported in aeroallergen SLIT [52]. EPIT aims to achieve desensitization via the skin. An adhesive patch is placed daily to the back or inner arm. The dose is fixed (for most studies 250mcg is used for peanut) and significantly lower than OIT doses. The safety profile for EPIT is favorable, with mild local reactions at the patch site observed in over 90% of patients receiving treatment and mild non-local reactions in less than 20% of subjects. Systemic reactions are rare with EPIT [53–56]. Patients treated successfully with the peanut patch for 12 months showed a 35.3% response rate for desensitization [57]. Additionally, they estimated a relative risk reduction of 73.2 to 78.4% when consuming peanut-contaminated packaged food products [58]. A recently published follow-up report of longer-term EPIT (involving an additional two years of treatment after reaching maintenance) in peanut-allergic children demonstrated sustained clinical benefit with high compliance and low discontinuation rates due to adverse events [57]. Most research trials have examined efficacy in children between the ages of 4–17 years old. However, OIT appears to work in the very young age group also. A study by Vickery et al. showed that 78% of 40 preschool children achieved SU following an average of 29 months of peanut OIT [59]. Follow-up at 5 years post-treatment showed the majority of children continuing dietary peanut consumption, with 55% ingesting more than 1000mg peanut protein without reactions [60].

Changes in QoL post-immunotherapy have been reported for both OIT and EPIT. A randomized-controlled trial of peanut OIT in 99 children from the UK showed significant QoL improvement following successful desensitization [61]. Blumchen et al. also reported significant improvement in QoL in 62 children undergoing low-dose peanut OIT in a multicenter, double-blind, randomized placebo-controlled trial [62]. Epstein-Rigbi et al. showed that the QoL of 191 children with food allergy improved significantly upon reaching OIT maintenance, with additional improvement 6 months later [63]. The caregivers’ QoL also improved in a trial of multi-allergen food immunotherapy [64, 65]. A recent manuscript reported that EPIT treatment was observed to be associated with significant global and domain-specific food allergy quality of life improvement largely driven by increases in eliciting dose, in children with peanut allergy [66].

In summary, the main benefit of immunotherapy treatment is protection from accidental exposures, whereas the main risk reflects allergic reactions (including anaphylaxis). Based on a mathematical modeling of risk, a rise in threshold from less than 100 mg of peanut protein to 300 mg post immunotherapy has been shown to reduce the risk of experiencing an allergic reaction by more than 95% for various food products with potential peanut contamination [67]. Although OIT, SLIT, and EPIT are not currently providing a cure for food allergy, protection from accidental food allergen exposure is observed in children who continue on regular therapy, whereas sustained unresponsiveness after discontinuation of treatment is much less common. Future research directions include improving safety and efficacy of food immunotherapy and examining patients’ goals for therapy and treatment outcomes. The role of immunomodulators alongside peanut OIT is also under investigation.

Recently, in the USA, the first drug for peanut OIT was approved, by the FDA and European Medicines Agency, paving the way to more commercial treatments in the future. The landscape of food allergies is changing rapidly, and patients will soon have options available in addition to traditional avoidance strategies. This raises the question of appropriate decision-making, and in nut allergy management, decisions are likely not straightforward. The patient will be faced with a variety of treatment options and no clear indication of the “best choice.” There are many potential management paths, each having trade-offs, and parents (and patients) often have very particular preferences for care [68]. The process of how to choose a particular option becomes a discussion where the clinician and the patient have to jointly review the medical evidence, but also the patient’s preference for balancing particular attributes of the treatment (both positive and negative) [68]. By working together, they ensure achievement of the best possible outcome.

## Conclusions

**Fig. 1:**
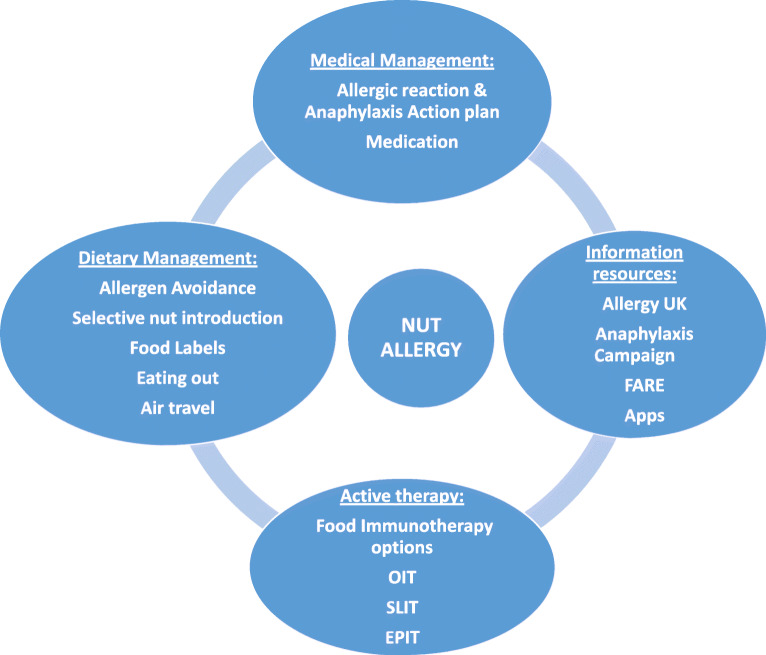
Nut allergy management comprises dietary, medical and active therapy management such as oral immunotherapy (OIT), sublingual immunotherapy (SLIT) or epicutaneous immunotherapy (EPIT), in combination with education and resources for patients and the wider community.

The management of nut allergy involves a combination of dietary and medical management coupled with education and information for patients, parents, and the wider community; it has evolved over time, with new evidence promoting more active approaches, such as selective nut introduction and nut immunotherapy (see Fig. 1). Multiple novel resources are now available to patients and families including apps and various, respectable support organizations. The physician maintains a key role in educating nut-allergic patients in the management of their disease. Dietetic input is also key and provides a much needed additional support in navigating daily life activities.
